# The Burden of Carbohydrates in Health and Disease

**DOI:** 10.3390/nu14183809

**Published:** 2022-09-15

**Authors:** Vicente Javier Clemente-Suárez, Juan Mielgo-Ayuso, Alexandra Martín-Rodríguez, Domingo Jesús Ramos-Campo, Laura Redondo-Flórez, Jose Francisco Tornero-Aguilera

**Affiliations:** 1 Faculty of Sports Sciences, Universidad Europea de Madrid, Tajo Street, s/n, 28670 Madrid, Spain; 2 Grupo de Investigación en Cultura, Educación y Sociedad, Universidad de la Costa, Barranquilla 080002, Colombia; 3 Studies Centre in Applied Combat (CESCA), 45007 Toledo, Spain; 4 Department of Health Sciences, Faculty of Health Sciences, University of Burgos, 09001 Burgos, Spain; 5 LFE Research Group, Department of Health and Human Performance, Faculty of Physical Activity and Sport Science-INEF, Universidad Politécnica de Madrid, 28670 Madrid, Spain; 6 Department of Health Sciences, Faculty of Biomedical and Health Sciences, Universidad Europea de Madrid, C/Tajo, s/n, Villaviciosa de Odón, 28670 Madrid, Spain

**Keywords:** metabolic disease, cancer, gut microbiome, asthma, nutrition, carbohydrates

## Abstract

Foods high in carbohydrates are an important part of a healthy diet, since they provide the body with glucose to support bodily functions and physical activity. However, the abusive consumption of refined, simple, and low-quality carbohydrates has a direct implication on the physical and mental pathophysiology. Then, carbohydrate consumption is postulated as a crucial factor in the development of the main Western diseases of the 21st century. We conducted this narrative critical review using MedLine (Pubmed), Cochrane (Wiley), Embase, and CinAhl databases with the MeSH-compliant keywords: carbohydrates and evolution, development, phylogenetic, GUT, microbiota, stress, metabolic health, consumption behaviors, metabolic disease, cardiovascular disease, mental disease, anxiety, depression, cancer, chronic kidney failure, allergies, and asthma in order to analyze the impact of carbohydrates on health. Evidence suggests that carbohydrates, especially fiber, are beneficial for the well-being and growth of gut microorganisms and consequently for the host in this symbiotic relationship, producing microbial alterations a negative effect on mental health and different organic systems. In addition, evidence suggests a negative impact of simple carbohydrates and refined carbohydrates on mood categories, including alertness and tiredness, reinforcing a vicious circle. Regarding physical health, sugar intake can affect the development and prognosis of metabolic disease, as an uncontrolled intake of refined carbohydrates puts individuals at risk of developing metabolic syndrome and subsequently developing metabolic disease.

## 1. Introduction

The progress of science in recent decades has made possible a social development without equivalence in the history of the human being. Advances in medical science have made it possible to eradicate diseases that were the cause of certain death a few years ago [1]. However, in the last decades, the cases of a new group of diseases that have not been identified throughout the evolution of the human being have increased [2]. We are talking about the diseases of civilization, where obesity and type II diabetes have positioned themselves as a true global pandemic [3,4]. The analysis of the triggering factors of these diseases has an important behavioral basis. The combination of inactivity and changes in nutritional intake behaviors, together with the current psychosocial context that is so exciting, have made this great increase in these pathologies possible [5]. Within nutritional changes, the role of carbohydrates is essential in understanding the development of these pathologies and in proposing efficient and effective interventions. For this reason, this narrative review is proposed with the aim of analyzing the impact of carbohydrates on health.

**Figure 1:**
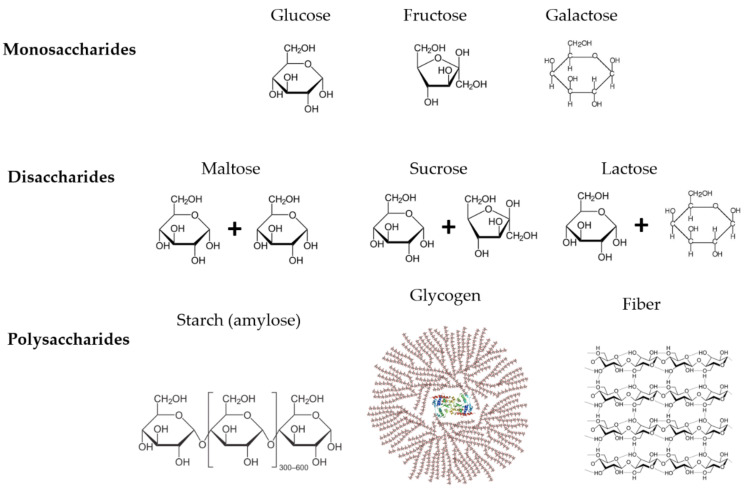
Chemical composition of carbohydrates.

Carbohydrates can be divided into two main types: simple and complex. Simple carbohydrates are made up of just one or two sugar units, whereas complex carbohydrates are made up of many sugar units. Between simple carbohydrates, we found the monosaccharides composed of one sugar (glucose, fructose, and galactose), disaccharides composed of two sugars (maltose, sucrose, and lactose), and complex carbohydrates with many sugars (starch, glycogen, and fiber) (Figure 1).

The rapid progress of science, the multiple areas of specialization, and the new scientific results that continue to delve into increasingly specific fields of knowledge often mean that the general perspective of a problem or an area of knowledge and the interrelationships with other areas are missed. The structuring of current knowledge and multi-area relationships with the integration of academic tools such as narrative reviews allow an efficient transmission of knowledge at a higher education institution level, as well as a better understanding of the study area for the research groups and academic professionals, enabling the implementation of new, more efficient, and multi-area research.

## 2. Methods

To reach the study aim, a narrative critical review was performed, analyzing primary sources such as academic research and secondary sources such as databases, web pages, and bibliographic indexes, in line with procedures used by previous critical narrative reviews [6,7,8,9,10]. We used MedLine (Pubmed), Cochrane (Wiley), Embase, and CinAhl databases, using the MeSH-compliant keywords carbohydrates and evolution, development, phylogenetic, GUT, microbiota, stress, metabolic health, consumption behaviors, metabolic disease, cardiovascular disease, mental disease, anxiety, depression, cancer, chronic kidney failure, allergies, and asthma. We used manuscripts published between 1 January 2012 and 15 June 2022, although previous studies were included to explain some information in several points of the review. We included manuscripts in both the English and the Spanish languages. We used the following exclusion criteria in line with previous reviews [11,12]: (i) research outside the period analyzed, (ii) presented topics out of the review scope, (iii) unpublished studies, books, conference proceedings, abstracts, and PhD dissertations. We used all the studies that met the scientific methodological standards and that had implications with any of the subsections of the present review. The treatment of the information was performed by all the authors of the review and, finally, the articles selected were discussed for writing the present review.

## 3. Human Evolution and Carbohydrates Consumption

The global increase in the incidence of obesity and diet-related metabolic diseases has raised the question of the underlying reasons for this problem [2]. Thus, the causes must be related to profound environmental changes, followed by changes in human behavior, especially dietary behavior. These environmental changes and behavioral modifications have led to a disruption of the balance between man’s genetic structure and his environment [13]. As a result, there has been an increased interest in the diets of our past, leading to a greater study of the foods consumed during that time and the role that food has played in human evolution [14,15]. In this regard, although it has been argued that the transition from a fibrous plant-based diet to a predominantly meat-based diet is one of the main drivers of evolutionary change [16], it is difficult to reconstruct an accurate picture of the diet of our ancestors.

However, carbohydrates from plants and meat are considered to have been necessary and complementary dietary components in the evolution of hominins into modern humans [13]. Thus, starch from plant foods was essential to meet the increased metabolic demands of a growing brain and increased aerobic capacity [17], which may have been an important evolutionary advantage to omnivorous hominins in the middle and late Pleistocene period. In addition, it has been proposed that the shift from a high-volume low-energy diet to a low-volume high-energy diet has led to several key changes in hominin morphology associated with the emergence of *H. erectus*, such as a substantial increase in height and body weight, a reduction in tooth size, and an increase in brain size [16,18,19]. Similarly, there was variation in the copy number of the salivary amylase gene (AMY1), which initiates starch digestion in the mouth and is related to blood glucose levels and carbohydrate digestion [13].

Another transformational event in human evolution was that humans are the only species to cook food [20]. Although the exact time at which cooking became widespread is not known, it is thought to have been long enough for biological adaptations to occur, such as a reduction in tooth size and a reduced ability to digest raw and fibrous foods due to a reduction in gut size [21]. In this regard, it is believed that the cooking of starchy plant foods co-evolved with an increase in salivary amylase activity. Without cooking, the consumption of starchy plant foods is unlikely to have met the high glucose demands observed in modern humans [20]. Similarly, the increased accessibility of starch to amylases through cooking would, in turn, have led to a greater advantage for high levels of salivary amylase expression, particularly in infants [21]. Thus, the regular consumption of starch-rich plant foods provides a consistent explanation for the energy needs of the developing brain during the late Pliocene and early Pleistocene periods, while the refinement of cooking and the concomitant increase in salivary amylase expression explains how the rapid increase in brain size from the middle Pleistocene period onwards was energetically affordable [21].

Plants produce a wide range of carbohydrates that serve as energy reserves for structural functions. Starch constitutes up to 80% of the dry weight of some edible roots and tubers and, if left intact in the soil, they remain stable and can be harvested for months when needed. Thus, the ability to utilize starch-rich roots and tubers in early hominin diets is seen as a potentially crucial step in differentiating early australopithecines from other hominins and in enabling expansion into new habitats [20]. In this regard, and despite the fact that evidence of plant foods rarely survives, making it difficult to reconstruct their diet, it is likely that the gradual replacement of fibrous plants with more energy-efficient plant foods, including starchy tubers, led to a reduction in gut size [22]. In addition, it has been proposed that, although meat may have been their preferred food, the energy expenditure required to obtain it may have been much greater than that used to collect tubers from a known and secure source [23].

### Nutritional Needs of Today’s Human Beings

The human brain alone accounts for 20–25% of adult basal metabolic expenditure [24]. In addition to the high energy demands of the brain, approximately 170 g/day of glucose is required for our brain, kidney marrow, red blood cells, and reproductive tissues to function properly. This glucose is mainly supplied by dietary carbohydrate intake, although it can also be obtained from gluconeogenesis or from propionate absorbed from the intestinal fermentation of dietary carbohydrates [25]. This high glucose/energy demand has meant that from the Paleolithic period onwards (~60,000 years ago), overexploitation of resources, climatic changes, and population growth led to a more diverse dietary pattern, which helped to establish the genomic structure of modern man [26]. Nevertheless, the emergence of modern agriculture and animal husbandry, and more recently the industrial revolution, has modified the diet without parallel changes in genetic structure, a phenomenon known as evolutionary discordance. Thus, the food groups with the greatest changes have been an increase in the consumption of cereals and refined sugars, as well as dairy products, refined vegetable oils, and fatty meats [13]. These changes have been associated with lower energy expenditure in urban communities. Thus, the health risks associated with these dietary changes are at the root of the epidemic of nutrition-related chronic diseases [26]. It will be necessary to adopt changes that bring us back to the Paleolithic diet, with the advantage that extensive food technology is now available.

## 4. Gut and Carbohydrates

The human body is made up of bacteria, viruses, protozoa, fungi, and archaea that live in balance, generating perfect body homeostasis, with a ratio of human: bacteria cells in the body close to 1:1, with bacteria cells being higher [27]. The above form the microbial tissues, which are found in the walls of tissues and are as important as the oral walls, nasopharynx, pulmonary, and gut [28]. In this line, the gastrointestinal tract represents one of the largest interfaces between the human body and the environment, exposing the human organism to a vast number of microorganisms throughout an average lifetime, which supposes a threat to gastrointestinal integrity [29]. To protect the gastrointestinal tract, a symbiotic relationship is established with the humans as hosts. This relationship has evolved for thousands of years, forming a mutually beneficial relationship such as shaping the intestinal epithelium, harvesting energy, protecting against pathogens, and regulating host immunity [30,31,32,33].

Furthermore, gut microbiota provide essential capacities for the fermentation of non-digestible substrates (dietary fibers and endogenous intestinal mucus), thus supporting the growth of specialized species that produce short chain fatty acids (SCFAs) and gases [34]. The major SCFAs produced are: butyrate, which is the main energy source for human colonocytes and which activates intestinal gluconeogenesis [35]; propionate, which is transferred to the liver where it regulates gluconeogenesis and satiety signaling; acetate, which is the most abundant and is, essential for bacterial growth and is a participant in cholesterol metabolism and lipogenesis [36]. Additionally, a higher production of SCFAs correlates with lower diet-induced obesity and with reduced insulin resistance [37]. In this way, low fiber intake reduces the production of SCFAs and shifts gastrointestinal microbiota metabolism to use less favorable nutrients, leading to the production of potentially detrimental metabolites [38]. Furthermore, the low fiber Western diet degrades the colonic mucus barrier, increasing permeability and causing inflammation and an invasion of the microbiota by pathogens [39], thus suggesting a high importance of having a diet rich in fiber [40]. However, a major increase in dietary fiber can temporarily reduce diversity, as the microbes that digest fiber become specifically enriched, which leads to a change in composition and reduces diversity through competitive interactions [37]. Therefore, fiber has an important role in the regulation of metabolism and in the prevention of chronic gastroenterological diseases [41,42,43] as there is clear evidence that fiber and SCFAs generated from its fermentation are extremely beneficial for the host [31]. Dietary fiber comprises plant-based carbohydrates that can only be metabolized by certain species of gut microbiota through anaerobic fermentation producing SCFAs [44]. Dietary fiber can be classified by the number of monomeric units. Fibers with 10 or more monomeric units include non-starch polysaccharides such as cellulose, hemicellulose, gums, pectin, mucilage, inulin, psyllium, and β-glucan [45], whereas fibers between 3 and 9 monomeric units are known as resistant oligosaccharides and include galacto-oligosaccharide (GOS) and fructo-oligosaccharide (FOS) [46]. However, the specific effects of fibers on the host depend on its physiochemical properties that are highly different between fiber types [47]: solubility (dissolution capabilities in water), viscosity (ability of fiber to form a gel-like consistency in water), and fermentation (degree to which they can be metabolized by the microbiota) [48].

Furthermore, dietary fiber can have a major impact on the composition, diversity, and richness of the microbiome [49]. Thus, individuals from industrialized countries, who normally consume a variation of the Western diet high in saturated fat and low in dietary fiber, have a higher abundance of microbial species linked to vast consumption of lipids and amino acids [50,51] (*Bacteroides, Bifidobacterium, Ruminococcus, Faecalibatcerium, Alistipies, Bilophila and Blautia*) [52,53,54,55,56], while non-industrialized populations have an abundance of the *Prevotella* species related to a high dietary fiber consumption [50]. As such, high fiber consumption in combination with the specific species of microbes able to ferment it will lead to a range of health benefits for the host [57].

Further on, the fraction of the diet reaching the large intestine is largely composed of complex polysaccharides resistant to human digestive enzymes. In addition, monosaccharides and disaccharides not fully absorbed in the upper gastrointestinal tract are used by the microorganism of the microbiota for its own metabolism [58]. Complex polysaccharides reaching the large intestine are resistant starch which cannot be degraded by amylase and glucoamylase in the upper tract like normal starch. Non-starch polysaccharides, including plant cell wall polysaccharides such as cellulose and hemicellulose organic polymer with polyaromatic structure lignin, which forms part of the cell walls of plants, and storage polysaccharides such as pectin and oligosaccharides are then fermented by microbiota in the large intestine [59], whereas monosaccharides and disaccharides that reach the lower gastrointestinal tract are lactose, fructose, and sugar alcohols (sorbitol, lactitol, and other polyols), which reach the lower tract due to intestinal malabsorption or overfeeding of these sugars, widely used for the formulation of processed foods or beverages [60].

In this way, carbohydrate availability can shape the whole microbial community as they impact fibrolytic communities and primary degraders and then affect all communities connected to them in a cascading manner [61]. Showing how through diet the gut microbiota of an individual can be affected even within days of a diet change [62]. Furthermore, resistant starches have been shown to enrich specific bacterial groups in some individuals depending on the carbohydrate’s chemical structure and the microbes’ enzymatic capacity to access them [63], with specific species becoming enriched to constitute more than 30% of the microbiota [64]. Thus, a diverse microbiota is synonymous with health and the ingest of microbiota accessible carbohydrates is seen as beneficial for the well-being and growth of these microorganisms and consequently for the host in this symbiotic relationship.

## 5. The Role of Carbohydrate Consumption in Behaviors

In recent decades, the consumption of carbohydrate-rich foods, and especially refined sugars, has increased exponentially because their consumption is associated with energy-boosting intake to combat fatigue and promote a sense of well-being [65]. Because their main ingredient is sugar, research has focused on understanding how these foods, and carbohydrates (CHO) in general, might promote the adequate cognitive status and emotional well-being [66].

In this regard, research has suggested that CHO intake may have mood-enhancing properties. It has been observed that, compared with healthy populations, individuals suffering from, for example, seasonal affective disorder or depression tend to increase their daily consumption of CHO-rich foods and beverages [67]. However, other recent studies have hinted that, in addition to the metabolic health problems associated with high sugar consumption [68,69], it may have adverse effects on psychological well-being, even leading to higher rates of depression in the long term [70,71]. On account of these controversial findings and the increasing trend of foods rich in refined sugars, knowing the real mental and physical health consequences of sugar consumption is of high importance.

The rationale for the claim that CHOs improve mental health and mood has a strong physiological basis. In this regard, although CHOs are used by the brain as a source of energy and as structural and functional components, other mechanisms have been proposed by which dietary carbohydrates might influence cognitive function such as neurotransmitter synthesis and effects via insulin and/or cortisol or via peripheral mechanisms [72]. In addition, fluctuations in serotonin availability following CHO ingestion [36,73] have been suggested to underlie the etiology of several mood disorders, such as depression, mania, seasonal affective disorders, anxiety, and aggression [74,75].

Along these lines, the intake of CHO-rich foods has been shown to have a protective effect against increased feelings of depression and reduced feelings of performance-related vigor [76,77]. Similarly, CHO-rich foods have been shown to reduce feelings of fatigue when compared with protein-rich foods [78]. Furthermore, while consuming low-CHO diets over long periods of time increases depression, fatigue, stress, and anger [79], consumption of CHO-rich diets may produce a reduced stress response [80], suggesting that CHOs may have a protective effect against stress and depression [67,81]. Similarly, the amount of CHO ingested daily has been negatively associated with rates of depression [82]. Researchers have proposed that, because of the strong relationship between CHO-rich foods, serotonin, and mood, people with mood or affective disorders may use CHO foods as “comfort foods” to lift their mood [83].

Despite the appeal of the serotonergic hypothesis and the literature documenting the effects of CHOs on various mood characteristics, studies examining interactions between CHOs and mood have produced inconsistent results. According to a growing body of empirical evidence from the past three decades, carbohydrate intake does not appear to significantly increase overall effect or subjective mood and may have negative effects on mood [84]. Researchers have questioned the validity of the effects of CHO on mood and have pointed out the complexity of the data [85,86,87]. The effects of CHO administration on cognition are potent and well-established [66,88], in contrast to the less reliable effects on mood. This difference could be explained by a variety of variables, including the different approaches used by studies to assess interactions between CHOs and mood.

Overall, the data from the various studies do not support the supposed CHO–mood link [84]. Even the evidence suggests a negative impact of carbohydrates on mood categories, including alertness and tiredness. These findings can inform public health policies aimed at reducing sugar consumption and promoting healthier alternatives, as well as increasing public awareness of the negative impacts of sugar consumption.

## 6. Carbohydrates and Metabolic Disease

Metabolic disorders develop when the normal metabolism of macronutrients (protein, lipids, and carbohydrates) is disrupted [89]. The metabolic syndrome is a combination of five risk factors that exponentially increase the risk of developing metabolic diseases such as atherosclerotic cardiovascular disease and type 2 diabetes, the most common causes of death globally [90,91]. Furthermore, the most important behavioral risks for the development of metabolic diseases are obesity, physical inactivity, and dietary habits characterized by a high consumption of sugars, fat, and salt and a low consumption of polyunsaturated fatty acids, vegetables, fruit, and fiber [92]. Furthermore, there are other metabolic disorders related to carbohydrate metabolism such as galactosemia, glycogen storage disease 1, Hunter syndrome, Hurler syndrome, mucopolysaccharidoses, mucolipidosis, and Pompe disease, among others [93].

Carbohydrates compose more than 50% of daily energy intake, as such the quality and source of theses carbohydrates matters as ingesting refined grains is associated with increased risks of metabolic disease [94], while whole grain intake is associated with a reduced risk of metabolic disease [95]. Additionally, the glycemic index and load are other factors to account for, as a higher dietary glycemic index or load is associated with an increased risk of type 2 diabetes [96] and other diseases, whereas low glycemic index diets are associated with a lower risk of developing metabolic diseases [97]. Thus, insulin and leptin signaling cascades and the central nervous system are strongly involved in key metabolic signaling pathways for metabolic diseases [98,99]. Insulin is fundamental for maintaining glucose homeostasis in post-absorptive and postprandial states, stimulating glucose uptake in insulin-sensitive tissues (muscle, fat, and liver). Hence, an impaired insulin regulation signifies a reduced ability of insulin-sensitive tissues to respond to insulin signaling on carbohydrate metabolism [100]. Moreover, impaired insulin regulation is considered the hallmark for metabolic abnormalities [101], as impaired insulin regulation results in hyperinsulinemia and hyperglycemia, producing a systemic inflammation associated with the development of several chronic diseases: type 2 diabetes, cardiovascular disease, and cancer [102,103].

Furthermore, current lifestyle and consumption of highly refined carbohydrate-dense food may have a role to play in metabolic disease development through appetite and satiety signaling disruption [104]. Thus, serotonergic and dopaminergic systems within the hypothalamus are considered as key regions for energy control in mammals [105]. Dopamine works as an inhibitor of food consumption and hyperphagia by establishing control over the number and duration of meals [106], whereas serotonin is known as a major regulator of feeding [107]. These systems may be affected by a high refined carbohydrate diet producing abnormal eating behaviors, resulting in increased visceral obesity and the development of metabolic diseases [108] or the worsening of its prognosis.

Currently, the use of drugs for metabolic disease treatment is limited due to the side effects [109]. For this reason, physical activity, weight control, and diet are the best options for the treatment of these diseases [110,111,112]. Moreover, diet interventions including non-digestible carbohydrates (resistant starch and dietary fiber) have shown increased intestinal viscosity, fecal bulking, and production of SCFAs resulting in improved blood glucose, lipid, and insulin levels, reducing energy intake and promoting satiety. However, these effects are different depending on the type, source, dose, and duration of the intake [113,114].

Summarizing, carbohydrate intake can affect the development and prognosis of metabolic disease as an uncontrolled intake of refined carbohydrates and puts individuals at risk of developing metabolic syndrome and subsequently developing metabolic disease. Therefore, physical activity, together with weight control and a healthy diet with a carbohydrate intake composed by low glycemic foods and non-digestible carbohydrates, would be beneficial for the prevention and treatment of metabolic disease.

## 7. Carbohydrates and Cardiovascular Disease

Some evidence links CHO and mortality and cardiovascular disease incidence. A previous metanalytical cohort study (*n* = 272,216) found an association between low-CHO diet or low-CHO and high-protein diet and a significantly increased risk of all-cause mortality [115]. In the same study (*n* = 469,963 participants), the relationship between low-CHO diet or low-CHO and high-protein diet did not significantly increase the risk of cardiovascular disease incidence. However, another study [116] that analyzed the relationship between low-CHO diets and mortality (*n* = 462,934 participants) reported that people with the lowest carbohydrate intake (39% TDE) had the highest risk of cardiovascular disease, cancer, cerebrovascular disease, and overall mortality. Nevertheless, the possible reasons for the association between low-CHO ingestion and a higher risk of mortality are not well established yet. The most common hypothesis is related to the reduced intake of fruits, grains, and vegetables, with a concomitant increased intake of animal-based proteins with a CHO restriction diet, affecting the levels of dietary bioactive components (fiber, minerals, vitamins, etc.) [117].

On the other hand, high-carbohydrate dietary patterns are traditionally associated with low rates of coronary heart diseases [118]. However, it is hypothesized about the reason for the reduction of cardiovascular risk due to carbohydrate ingestion and it seems that some characteristics of this type of diet, with low fat (specifically saturated fat) and higher promotion of satiety, may be involved in the protection against obesity or overweight [118]. In fact, it is possible that high amounts of carbohydrate diets play a role as a marker for some other protective factor that reduces the risk of cardiovascular diseases.

Actually, the sources of dietary carbohydrate are an essential factor likely associated with the clinical and physiological effects that can promote or impair health. In this way, in most countries, substantial amounts of total sugars are derived from sucrose or high-fructose corn syrup among other free sugars. These types of food, with a higher proportion in free sugars are energy dense and usually have a poor proportion of essential micronutrients [119] and may have clinical consequences when compared with carbohydrates derived from vegetables and fruits. This paradox in sources of dietary carbohydrates could be observed in some developing countries, where higher CHO ingestion is associated with lower economic status and lower quality CHO foods with higher glycemic index and refined carbohydrate [116] but also with a high proportion of sugars derived from vegetables and intact fruits in people with higher economic status.

During the last decades, results from CHO diet studies focused on the cardioprotective role of cereal grains and dietary fiber have been published. Specifically, whole grains have growth interest. Whole grains are characterized by their high composition in dietary fiber, resistant starch, phytoestrogens, micronutrients (i.e., vitamins and folic acid), and antioxidants [120]. In this way, a previous meta-analysis found a reduction in risk of cardiovascular disease, stroke, and coronary heart disease if patients consume a whole grain intake (90 g/day) [121]. These results agree with other two meta-analysis that found that higher consumers of whole grains (48–80 g/day) have a 21% lower risk of cardiovascular disease compared with lower consumers of whole grains [95,122]. Associations between whole grain consumption and risk factors for coronary heart disease have also been reported in other studies that found a beneficial relationship between whole grain consumption and the risk of total mortality and incidence of coronary artery disease but not the risk of ischemic stroke [123]. Moreover, there is evidence of an inverse relationship between total cholesterol, low-density lipoprotein (LDL) cholesterol, and body mass index with whole grain consumption [124]. However, the Cochrane review [125] found no evidence from randomized controlled trials (RCTs) of the effect of whole grain diets or foods on cardiovascular mortality or events; however, evidence on the effect of whole grain on cardiovascular risk factors such as lipids or blood pressure is currently available.

Regarding the effect of a CHO diet on cardiovascular risk factors, some previous studies have analyzed the different proportion of CHO diets on weight loss and on determinants of energy balance and body weight. Specifically, diets with a low proportion of CHO increase energy expenditure, perhaps due to the changes in catecholamines and thyroid hormones, although the mechanisms contributing to this effect are incompletely understood [117]. Moreover, low and very low-CHO diets reduce hunger and appetite due to the changes in gastrointestinal hormones [126]. Nonetheless, the effect of a CHO-restriction diet on weight loss in an intervention longer than six months promotes the same change in weight loss compared with a calorie-restricted low-fat diet [127]. However, short-term (<6 months) low and very low-CHO diets produce a greater weight loss than hypocaloric high-CHO low fat diets [128,129]. Interestingly, diets with CHO restriction seem to produce a greater loss of lean body mass than other macronutrient balanced hypocaloric diets [130]. Therefore, higher protein intake is recommended in diets with CHO restriction to prevent lean body mass reduction during weight loss programs [131].

Blood lipid markers are also affected by carbohydrate ingestion. In this regard, previous meta-analysis found a decrease in total cholesterol and LDL in diets with low or very low intake of CHO [132,133]. In addition, a decrease in triglycerides [134] and a short-term increase in HDL [128,134] are produced in diets with low carbohydrate ingestion. With respect to blood glucose, another cardiovascular risk factor, some studies demonstrate that the amount of carbohydrates of the diet does not modify fasted blood glucose or the insulin level [128,135]. These meta-analysis studies found that low-CHO proportion diets do not reduce these two outcomes any more than high-CHO strategies. However, diets with a lower proportion of CHO produce a reduction in the pharmacological therapy in diabetic patients [136,137], which can increase the risk of cardiovascular diseases and mortality [138]. Lastly, the results of the effect of carbohydrate ingestion on blood pressure are inconsistent. Some previous systematic reviews with meta-analysis [134,135] found no difference in systolic and diastolic blood pressure between diets with high- or low-CHO intake. However, another meta-analysis reported no difference in SBP but found a significant difference in DBP between diet groups, in favor of low-CHO diets [129].

On the other hand, previous evidence suggests that many nutrients and diet components can modulate inflammation both acutely and chronically [139]. In such a way, it is previously demonstrated that a chronic low-grade inflammatory state is a pathological feature of some chronic diseases such as cardiovascular diseases, metabolic syndrome, or type 2 diabetes mellitus [140]. Regarding carbohydrates and inflammation markers, high-glycemic index carbohydrates increase inflammation markers such as nuclear factor-kB activation and nuclear factor-kB binding in mononuclear cells [141]. In addition, low glycemic load and high wholegrain diets may have a protective effect against systemic inflammation [142]. Moreover, an inverse relationship between C reactive protein and dietary fiber has been previously published [143]. Therefore, a healthy pattern based on low glycemic index carbohydrate, characterized by reduced postprandial glycemia can reduce the concentrations of inflammation markers.

In summary, evidence shows that the nature of the carbohydrate rather than the amount is the key factor in reducing the risk of cardiovascular disease or in improving the cardiovascular risk markers. In this way, carbohydrates derived from intact fruits and vegetables, legumes, whole grains, and overall low glycemic index carbohydrate diets are recommended to reduce the risk of cardiovascular diseases due to the dietary fiber and other potentially cardioprotective components. Some of these food resources, especially those with a high proportion of dietary fiber, can improve cardiovascular risk factors such as total cholesterol or low-density lipoprotein

## 8. Carbohydrates and Mental Diseases

In recent years, the relationship between diet and mental health has gained considerable interest. Although the scientific community has typically focused on observing dietary patterns in general, it has become necessary to examine specific macronutrients in isolation. The normal daily dietary intake of a person consists of 50 to 60% carbohydrates [144]. Thus, this caloric intake inserts the most outstanding impact on our organism. Regarding mental health, defaulted carbohydrate metabolism and abnormal brain function can result in various psychological as well as physiological disorders; depression and anxiety being the most common [145]. However, everything depends on the type of dietary carbohydrate [146]. Following the FAO and WHO recommendations, the bulk of carbohydrates consumed should be rich in dietary fiber and should have a low glycemic index (GI) [118]; considering this, its importance in mental health needs to be addressed.

At different stages of life, human beings change their carbohydrate consumption. From this point, glucose has received particular attention because it is the preferred and primary source of energy for the brain. Most studies in children show that the brain appears to be sensitive to short-term variation in the availability of glucose. In this line, researchers have demonstrated that the memory for spoken words in very young infants is enhanced after a glucose feed [147,148]. Furthermore, other studies investigated the role of sugars as reinforcement in early learning [149]. However, studies also showed that added sugar was not associated with creative thinking [150]. When adults are assessed, evidence suggests that higher GI and sugar intake were associated with an increased risk of incident depression in women [151], but an inverse link between glycemic load (GL) and mental disorders, depression, and psychological distress was demonstrated in other studies with adults and the elderly [152,153]. On the contrary, a controlled-feeding study carried out by Breymeyer et al. (2016) [154] showed the effect of high and low glycemic-load diets. After 4 weeks, compared with the low glycemic-load diet, the Profile of Moods State Questionnaire subscales including Fatigue–Inertia and Total Mood Disturbance scores were higher after the high glycemic load diet [155]. In this regard, this discordance is likely because the glycemic-load calculation includes the glycemic index of foods consumed as well as the total carbohydrate intake, and, as previously described, carbohydrate intake per se is not associated with depression risk. Therefore, these analyses suggest that an intake of added sugars and refined carbohydrates is positively associated with depression [156]. Apart from this, it is well established that the dietary intake of protein and carbohydrates plays an important role in the metabolic activity and taxonomic profile of the gut microbiota, which can influence depression and anxiety by modulating the stress reaction system and the inflammatory response [157]. In this regard, a high-fat/low-carbohydrate diet, regardless of the type of fat, decreases total bacteria [158]. However, fiber is important in shaping the microbial ecosystem and has the potential to improve the gut-brain axis [159]. Due to the gut microbiota playing a fundamental effect on gut inflammatory and immune responses, it is highly likely that the role of gut microbiota dysbiosis in the pathophysiology of depression is induced by immune and inflammation responses [160]. In this line, strong evidence indicates that a diet rich in vegetables, fruits, fibers, fermented foods, vitamins, probiotics, and polyunsaturated fatty acids helps maintain gut microbiota homeostasis and promotes positive mental health. In contrast, high-fat, high-carbohydrate, and ultra-processed foods are associated with gut dysbiosis, inflammation, and poor mental health [161]. Moreover, high-GI diets could also lead to insulin resistance, which has been associated with a pattern of volumetric and neurocognitive deficits, which are very similar to that found in individuals suffering from clinical depression [162]. Therefore, carbohydrate quality might be an important determinant of psychological health status.

In relation to the above, one of the main reasons related to diet and poorer mental health is the current obesogenic environment, with the wide availability of palatable foods such as saturated fats and refined grain and sugar products with higher GI that may drive addiction behaviors [163,164]. This tendency is a frequent cause of weight gain and can also be seen not only in patients who become fat when exposed to stress [165] but also in depression [166]. This is due to an optimal tryptophan availability to the brain which in response releases considerable amounts of serotonin in the blood, evoking a natural calming but short effect. Particularly, Oddy et al. (2009) [167], and also Kim et al.’s, research [168], proved that a diet containing plenty of sweetened beverages, soft drinks, snack foods, junk or fast foods, commonly known as the Western diet, ends up increasing the stress levels, psychological disturbances, and poor mental health. On the contrary, lower glycemic loads are found to be associated with improved mental health within different groups of the population. In contrast, high-GL diets can result in rapid late post-ingestive decreases in blood glucose with a tendency to hypoglycemia, which could be responsible for triggering central dysfunction and depression, thus possibly contributing to the higher prevalence of depression [153].

An important question arises about the perfect combination of CHO and macro- and micronutrients. Focusing on CHO intakes, compared with people who consume a diet based on proteins, it has been demonstrated that balanced diets with sufficient complex carbohydrates improved mood and cognition and people on such a diet were calmer and less anxious [169]. The Mediterranean Diet (MD) is among the most balanced diets since it provides plenty of protein, carbohydrates, fats, and relevant micronutrients. Additionally, it provides good control of glycemia, which can reduce the risk of diabetic complications in the short-, medium-, and long-term [170] and can promote better mental health. Compared with the Western diet [171], adherence to the MD, consisting of fresh plant-based foods, moderate in fish, olive oil, and eggs, and low in saturated fat and red meat, is also associated with healthier gut microbiota and consequently better brain function [172]. Particularly, fiber from vegetables, fruits, legumes, and whole grains might have a beneficial role in gut health and therefore in mental health [173,174]. Its anti-inflammatory effects can be transmitted to the brain via pathways involving direct central nervous system signaling and immune system activation [173]. Due to this, not only neurocognitive disorders may have been significantly reduced but also depression, anxiety, mood disturbances, and stress [175]. To conclude, carbohydrates, when taken in an adequate amount and in the right balance in the diet, can sustain good mental health; however, more research is needed in this regard.

## 9. Carbohydrates and Cancer

It has been largely described how carbohydrates may have an important effect on cancer development due to their capability of enhancing glycolysis, the most relevant source of energy that tumor cells have. Additionally, carbohydrates may modulate different pathways which could enhance cancer promotion as well as cell proliferation and migration.

A hundred years ago, in 1920, Otto H. Warburg elucidated how tumor cells mainly develop an aerobic glycolysis as a metabolic source [176]. In the present, this process is also known as the Warburg effect, described as an aggressive phenotype presented by most cancer cells. Nowadays, it has been highlighted that the Warburg effect is caused by metabolic reprogramming in tumoral cells [176]. Currently, it has been pointed out by several authors that glycolysis has been found increased in tumoral cells [177], being it is the main process assessed to obtain adenosine triphosphate (ATP) as an energy key factor. As a consequence of this basic metabolic process, glycolytic metabolites play an important role in the novo synthesis of cell proliferation development, in which nucleotides, lipids, and amino acids are produced [178,179,180]. Furthermore, the tricarboxylic acid cycle (Krebs cycle) also has been recently reported as an essential anabolic source of tumor progress, [180,181,182] since pyruvate carboxylase, the enzyme which is responsible for the transformation of oxaloacetate from pyruvate, the last metabolite of glycolysis, has been pointed out as an important key factor in tumor advance [183,184,185]. Consequently, both glycolysis and the tricarboxylic acid cycle are considered in the present day as the most relevant metabolic sources that enhance tumor growth [180,186].

Regarding the tumoral pathways, the previous literature also described how high glucose values may have a negative effect in cancer prognoses. Thus, hyperglycemia produced by the consumption of a large amount of simple carbohydrates has been related to a worse efficacy in cancer treatment [187,188,189,190,191], since these cancer cells would take advantage of these nutrients, improving their quick expansion and promoting their growth and proliferation. Moreover, independently of the origin of high glucose levels (dietary origin or diabetes mellitus), it has been pointed out how hyperglycemia may modulate angiogenesis, improving vascular endothelium proliferation as well as through de-inhibition of anti-angiogenic factors [192]. Thus, it was reported in previous research that increased glucose levels may upregulate the vascular endothelial growth factor (VEGF) and vascular endothelial growth factor receptor (VEGFR) expression in different kinds of cancers, which are involved in angiogenesis processes, enhancing cell proliferation and survival. Finally, previous authors identified the metastatic potential of hyperglycemia in tumoral cells, since epithelial cells suffer an epithelial–mesenchymal transition that allows them to adjust themselves to the surrounding environment, promoting their motility. Then, high glucose levels may influence the upregulation of transcription factors, which involves EMT-related genes. As a result, migration and metastasis may be encouraged through hyperglycemia [192,193].

With the aim to improve cancer prognosis, several dietary modifications have been proposed in the previous literature. Firstly, fasting and autophagy have been proposed as useful tools that may improve different cancer prognoses, since previous researchers proposed how autophagy may participate in cancer cell death, improving apoptosis mechanisms. Moreover, fasting enhances autophagy, also improving immunological and treatment response [194,195]. This fact could be explained through an obvious mechanism. Proliferation and cell growth would take place in a wealthy nutrient environment. Then, in absence of nutrients, glycolysis, as well as nucleotide and protein synthesis, would be inhibited, compromising tumoral development [196]. Additionally, to modulate glucose levels, two different kinds of diets were proposed, including low and very low carbohydrate diets. Regarding the very low carbohydrate intake, a 20–40% calorie restriction was related with a late development of tumoral events [197]. Different mechanisms explain the benefits of calorie restriction in cancer development, including raised insulin sensitivity and angiogenesis inhibition [198,199], as well as an increase in DNA repair processes, a raise in the apoptosis mechanism triggering in a damaged cell elimination and autophagy promotion [200]. Regarding the low carbohydrate intake, the ketogenic diet has been elucidated by its potential benefits in decreasing cancer progression. Thus, in a study conducted in brain glioma patients, a keto diet was applied, allowing 60 g of carbohydrates per day added to their basal treatment. Results in this research showed how the average survival time without illness progression was 5 weeks [201]. Moreover, in a study developed in female nurses who fed on a diet based on low carbohydrate intake, the results suggested that a ketogenic diet would be related to a lower risk of developing breast cancer with negative estrogenic receptors [202]. Additionally, the combination of a ketogenic diet and a paleo diet, which is based on the consumption of fruit, vegetables, fish, lean meat, and the avoidance of red meat, wholegrain products, and, in general, ultra-processed food, also yielded positive results in cancer progress. Then, the tumor growth inhibition was observed in a rectal cancer patient during the first five months, during which he strictly followed the diet [203].

The quality of carbohydrates also has been related to cancer events, since in a study conducted in Canada it was determined that breast cancer patients would have consumed high glycemic index carbohydrates, including simple sugars [204]. For example, in a study developed through 39 European countries, positive associations were found between refined sugar intake and colorectal cancer [205]. Additionally, higher colorectal cancer was associated with high glycemic index sugar consumption, raised glycemic load consumption, non-fiber carbohydrates, sucrose, and fructose [206]. These results may be explained by the fact that these high glycemic index and glycemic load diets trigger hyperglycemia and, consequently, raised insulin levels. These elevated insulin values promote the activity of insulin growth such as factor 1 (IGF-1), which has been associated with an increase in cell proliferation as well as an inhibition in the apoptosis process, enhancing cancer development [207,208]. Thus, considering different dietary patterns, those that include a low carbohydrate intake, such as fruit, vegetables, and legumes [209,210], would imply lower glycemic index and, consequently, a protective effect against colorectal cancer. Furthermore, in research conducted in the American Institute for Cancer Research, a relationship was reported between higher fiber intake and breast cancer survival [211]. Additionally, similar results were found in previous research, with positive associations between fiber consumption and a lower risk of breast cancer [212]. Moreover, several authors suggested how fruit and vegetables may have a positive effect in cancer, due to their higher fiber and complex carbohydrate content. The explanation of the protective effect of fiber and complex carbohydrates in cancer development may be explained through different factors. Firstly, complex carbohydrates may modulate insulin-like growth factor binding protein 3 (IGFBP-3) activity, which may interrupt insulin/IGF-1 axis, blocking cell proliferation and tumoral growth, as mentioned above. Secondly, complex carbohydrates and fiber could modulate gut microbiota, and consequently, their short chain fatty acid (SCFA) production, which may reduce inflammation, having a positive effect in cancer development. Finally, these complex carbohydrates and fiber may increase fecal excretion of carcinogens, improving their elimination and reducing their negative effects in an organism [213].

## 10. Carbohydrates and Chronic Kidney Failure

Chronic kidney disease (CKD) is a pathology that involves extensive kidney damage, where kidney filtration is largely compromised. According to diagnoses, it has been established that kidney failure is determined by two different situations. Firstly, it could be diagnosed as a maintained kidney damage for three months, verified by a kidney biopsy or biomarkers of kidney damage, accompanied or not by a reduction in the Glomerular Filtration Rate (GFR). Secondly, it could be diagnosed as a reduction in GFR under 60 mL/min per 1.73 m^2^ for three months, including or not kidney damage [214].

In addition, there are several pathologies which could compromise kidney filtration; diabetes could be considered one of them, which may have a higher impact in CKD. Thus, maintained raised glucose values that imply diabetes trigger microvascular changes in the kidney, which results in CKD [215]. These microvascular complications involve diverse physiopathology mechanisms. Initially, raised glucose levels are vastly filtered into the glomerulus, triggering a tubular hyper-reabsorption of sodium and glucose, which conduces to hypertrophy or complete atrophy, depending on disease severity, in both the glomerulus and the tubules. In late states, hyperglycemia could affect the glycocalyx of endothelial cells, promoting its opening and its destruction, which triggers protein loss and decreased peripheral micro-vessel density, leading to arteriole closure, which may develop atrophy as well. Additionally, this atrophy may be enhanced by cytokine activity, that is responsible for inflammation processes as well as tissue remodeling [216].

Regarding the important effect that hyperglycemia may have on CKD, several authors pointed out the significant effect that diet has in CKD prognoses. Then, it has been largely described how a raised carbohydrate intake may be related to increased CKD risk [217]. Nevertheless, not only carbohydrate consumption could be related to CKD, since high protein and low carbohydrate intake has been described as a CKD risk factor [218]. Regarding fat consumption, raised lipid ingestion and sugary diets have also been related to higher probabilities in CKD progression [219], whereas elevated polyunsaturated fatty acid consumption has been associated to lower CKD risk [220]. Moreover, recent researchers proposed that a high-carbohydrate and a low-fat diet was significantly associated with a raised risk of CKD [221].

Considering all these findings, it would be possible to consider lower carbohydrate diets as a useful tool that may contribute to reduce CKD advancement, since a high-fat content diet also has been pointed out as a predictor in chronic kidney failure. It is obvious to suppose that CKD implies the necessity of a reduction in protein intake, since glomerular filtration is compromised in chronic kidney failure. Then, raised protein consumption may damage renal function, since it would involve an extra effort developed by nephrons, due to higher protein size compared with other smaller molecules, which must cross vascular endothelial glycocalyx, compromising permeability [222]. Nevertheless, low-carbohydrate diets are usually related to high protein intake for obtaining energy, regardless of carbohydrates. To clarify this issue, recent research suggests that a very low carbohydrate diet does not compromise GFR [223]. Hence, considering results from this last research, it would be possible to consider a very low carbohydrate diet as a safe tool in response to a CKD patient’s nutrition management, using a protein intake around 1–1.4 g/kg of ideal body weight/day. Protein intake in this research could be considered as correct, since the authors applied protein consumption as recommended in the previous literature [224], in which diary protein intake was established between 0.8 g/kg body weight and 1.4 g/kg body weight. Then, in light of the previous literature, it could be defined that carbohydrates may have a great impact on chronic kidney failure development and progression.

Regarding specific carbohydrate intake, it has been largely pointed out how high-fiber diets may have a favorable impact on CKD. Then, previous researchers found a negative association between fiber consumption and the risk of developing CKD, since higher values in fiber intake were related to a lower risk in the CKD phenomenon [225]. It may be explained by the fact that fiber intake promotes the saccharolytic gut microbiota activity, which enhances the production of SCFAs. Then, these components, including butyrate and acetate, may be responsible for the reduction of metabolic acidosis, preserving GFR and decreasing CKD development [226]. Additionally, as mentioned above, SCFA has been associated with a reduction in inflammatory processes [213], a fact which could improve CKD control since inflammation could have a negative impact in chronic kidney failure. It has been elucidated by previous authors how inflammation could promote hypertension [227], an important key factor which could deteriorate CKD prognoses [228]. Then, if an inflammatory process persists, it will produce an endothelial dysfunction by modifying nitric oxide (NO) release from endothelial cells, triggering a reduction of NO bioavailability [229]. NO has an important function as a vasodilator [230], and its loss may lead to the development of hypertension [230], negatively affecting CKD. Regarding simple carbohydrates, it has been established how high sugar consumption may alter gut composition [231,232], causing gut dysbiosis and promoting the inflammatory effect mentioned above.

## 11. Carbohydrates and Allergies and Asthma

Asthma is a chronic respiratory condition characterized by inflammation of the airways. Although asthma is often believed to be a disorder localized to the lungs, current evidence indicates that it may represent a component of systemic airway disease involving the whole respiratory tract, and this is supported by the fact that asthma frequently coexists with other atopic disorders, particularly allergic rhinitis [233]. The prevalence of allergic rhinitis and other allergies has risen worldwide. In this regard, evidence demonstrates that there are associations between dietary factors and asthma risk and control in children and adults [234].

Therefore, it has also been suggested that disparities in asthma prevalence and mortality can be attributed to lower socioeconomic status and the greater health needs of minority groups [235]. The countries with the highest carbohydrate intake are all considered developing nations [235]. Among developed countries and the different types of diets, Spanish children were assessed by Calatayud-Sáez et al. (2015) [236], who concluded that the adoption of a traditional Mediterranean diet (plant-based foods, such as whole grains, vegetables, legumes, fruits, nuts, seeds, etc.) could contribute significantly to the improvement of patients diagnosed with childhood asthma [236]. Additionally, the diet could be a major contribution to the improvement of patients with recurring colds and frequent inflammatory complications [237]. Regarding the Western diet, current evidence is contradictory; some studies do not support an association between it and the incidence or prevalence of adult asthma but suggest a possible link between a Western diet pattern and adult asthma morbidity [238]. However, high-fat, sugar-, and salt-diet patterns based on eating pastry, chocolate and sweet desserts, candies, salty snacks, chips, fruit juices, soft drinks, and alcoholic beverages consumption as snacks were associated by other research with asthma prevalence [239]. Corroborating these results, it has been also suggested that a diet with a high intake of fat and simple sugars and a low intake of fruit, vegetables, and rice is associated with an increased risk of asthma in Taiwanese children [240]. Particularly, Wang’s study [241] stablished that the consumption of fast foods, especially hamburgers ≥ 3 times/week, was more likely to be associated with severe asthma and current wheeze compared with the consumption of 1–2 times/week.

The possible increase in weight due to the consumption of this type of diet and consequently having a higher BMI is also related to the incidence of asthma [242]. Not only the presence of an inflammatory component evoked by overweight and obesity may be related to asthma, but also intestinal permeability may be associated with other health alterations because of immunologic dysfunction. Particularly, asthma, according to the leaky gut theory, has been described as one of the allergic diseases caused by the activation of the immune system because of intestinal permeability [243]. Considering this, fructose is one of the key carbohydrates involved in the regulation of intestinal permeability and its overuse may cause harmful effects, such as tight junction protein dysfunction [244]. Consistent with this, several studies reinforce what has been stated above, suggesting that the high consumption of simple sugars used as sweeteners in different sugary beverages has been associated with asthma being more evident in the case of beverages containing high fructose, because its causes malabsorption, resulting in the intestinal formation of pro-inflammatory products between unabsorbed fructose and some dietary proteins that, after intestinal absorption, are associated with asthma [245]. This amount of high sugar can also affect the activity of a gut mucosal defense factor, intestinal alkaline phosphatase (IAP) [246]. Meanwhile, complex dietary carbohydrates (starches, glucans, fructans, and cellulose), and especially those from whole-grain products, have demonstrated an inverse association with inflammation [247].

Regarding fat diets, Kim and collaborators identified significant relationships between allergic rhinitis and high-fat diets; however, also with low-carbohydrate diets among Korean children [248]. Hence, the amount and the quality of carbohydrates play a crucial role in identifying which diet may be the optimal option. Likewise, with a high-carbohydrate diet based on cereals, rice, and nuts, the International Study of Asthma and Allergies in Childhood (ISAAC) Phase One study showed a strong negative association between its consumption and the prevalence of allergic diseases, even though it reflects a protective effect [249]. As mentioned above, the consumption of these complex carbohydrates typical of MD indicated a protective role against childhood asthma, but they also imply that the MD probably does not affect the development of allergies [250]. Concerning gender, asthma symptoms, asthma, and allergic sensitization were more prevalent in boys than in girls and were more prevalent in the south than in the north of France [251]. Nevertheless, other factors should be considered; allergic conditions such as allergic rhinitis, rhinoconjunctivitis, and eczema are prevalent among children and are associated with environmental tobacco and smoke, paracetamol use, antibiotic use, television watching, and outdoor and indoor air pollution [252]. The heterogeneity and limitations of the studies highlight the need for randomized controlled trials that will focus on the pediatric population and hopefully provide more robust evidence.

## 12. Carbohydrates: Actual Dietary Recommendations

Many guidelines for nutrient intake published by international organizations have stablished the quantitative and qualitative nutrient recommendations to prevent some nutrition-related diseases [253,254,255,256,257]. Regarding total carbohydrate intake, the different guidelines aforementioned recommend a total carbohydrate intake range from 40% [258] to 75% [259] of the total energy intake. These wide discrepancies are explained by the organization because the lower values of the range reflect the minimal physiological requirement, while the higher percentage is the optimal level for the decrease of the risk of disease. Specifically, the World Health Organization (WHO) proposed a goal of total carbohydrate intake between 55–75% of the total energy intake, which is the percentage of energy available after taking into account the fat and protein consumed [259].

On the other hand, in Europe, the European Food Safety Authority proposed a reference carbohydrate intake range of 45–60% of total energy intake [260], which is in agreement with the Nordic countries’ recommendations [261,262]. In addition, these intake ranges in dietary patterns are associated with a reduced risk of chronic diseases [263]. Other European countries’ guidelines do not agree with these previous recommendations. For example, Ireland recommends a total carbohydrate ingestion of 45–65% of total energy intake [264], while Spain proposed a range of 50–55% [256]. In the same way, the UK reference value of total carbohydrate ingestion is around 50% of total energy intake [265]. All of these recommendations want to meet energy needs and they fulfil the amount of glucose to brain metabolism, where a concomitant intake of a low amount of fat and saturated fat would decrease the metabolic risk factor in chronic diseases [263].

Recently, an update of the dietary guidelines for United States has been published, stablishing that carbohydrates make up 45% to 65% of total daily calories [253]. In addition, the recommended daily allowance based on the requirements for brain glucose utilization is established at 130 g/day in the same guideline. Australia and New Zealand proposed the same range of total carbohydrate intake (45–65% of the total energy intake), derived from low glycemic index food resources [257]. The reason for this range is that there is a higher risk of obesity with high-fat low-carbohydrate diets and a relationship between cardiovascular disease and high rates of carbohydrate intake (>65%).

With regard to sugars, some international organizations give a nutritional recommendation to reduce free sugar consumptions to levels <5% [257] or ≤10% [254] of the total energy intake. A higher proportion of free sugars’ ingestion increases the weight, type 2 diabetes, and the risk of dental caries [261,262]. In addition, there are several reports of a relationship between the increase of risk of various non-communicable diseases with sugar intake [263] that provide a statement to limit sugar consumption and foods with added sugars [266].

On the other hand, current guidelines have also published recommendations for fiber intakes. Overall, a value of about ≥25 or ≥30 g per day of fiber intake in adults has been given [257,258]. Specifically, some distinction in the amount of fiber ingestion was recommended according to the age and sex of the population. In this way, the European Food Safety Authority suggested a range between 10–21 g per day in children from 1 to 18 years [265]. In the same way, the reference values proposed by the United Kingdom are 15 g per day in children from 2–5 years, 20 g per day in children from 5–11 years, 25 g per day in children from 11–16 years, and 30 g per day in adolescents from 16–18 years and in adults [260]. Finally, the Nordic countries proposed lower values of fiber intake (25 g per day) in women than in men (30 g/day) [261,262]. These nutritional recommendations are based on the relationship between fiber intake and the lower risk of some diseases and health promotion. Some previous evidence has been reported on the protective effect of dietary fiber against type 2 diabetes, obesity, and cardiovascular disease [267] and against metabolic disease and the maintenance of correct bowel function [263]. Moreover, evidence on the protective effect of fiber intake against cancer (i.e., colorectal cancer) has also been reported [261,262]. Interestingly, the guidelines for fiber intake published by international organizations recommend that fiber should be obtained from a variety of food sources. For example, a WHO statement [259] recommends that the preferred sources of fiber are wholegrain cereals, fruits, and vegetables for providing the aforementioned ≥ 25 g/day.

With reference to whole grain, most of the dietary recommendations for whole grain ingestion is qualitative [265], promoting the intake of complex carbohydrates, particularly those from whole-grain cereals. The last update of the United States nutrition guidelines [253] has proposed some suggestions about whole grain daily intake. In this way, it is recommended that the healthy dietary patterns include whole grains and limit the intake of refined grains. Specifically, this guideline proposed that at least half of the total grains should be whole grains. For example, in adults (in a diet of 2000 kcal per day), from the 6 ounces of grains recommended per day, ≥3 ounce per day must be whole grains. Interestingly, 98% of the American population do not meet the recommendations for whole grains, but 74% exceed the limits for refined grains, because grains are consumed in the form of sodium and added sugars (e.g., ready-to-eat breakfast cereals) rather than the nutrient dense forms, which are foods more interesting in whole grain resources [253].

Concerning the glycemic index, previous reports found a positive relationship between a low glycemic index diet and health outcomes [257]. However, no specific advice about the glycemic index was given in most of the nutritional guidelines of the international organizations. In contrast, other organizations suggested that the high amount of carbohydrate ingested should be derived from low glycemic index sources [257]. However, this recommendation is controversial, because the evidence for the protective effect of the low glycemic index on chronic diseases is unclear [258].

In summary, nutrition guidelines stablished that carbohydrates make up 40% to 65% of total daily calories to fulfil body energy demands and to reduce the risk of some non-communicable diseases. The recommended daily intake of carbohydrates should be based on whole grains (at least half of total grain consumption) and limited for refined grains, limiting free sugar consumptions to levels < 5–10%. In addition, the high amount of carbohydrate ingested should be derived from low glycemic index sources and fiber intake should be higher than 25–30 g per day.

## 13. Practical Statements

After the critical revision of actual knowledge, we can propose the following practical statements.

- Increases in the incidence of obesity and diet-related metabolic diseases are related to profound environmental and behavioral changes, especially dietary behavior.
- The increase in the consumption of cereals and refined sugars, as well as dairy products, refined vegetable oils, and fatty meats, are at the root of the epidemic of nutrition-related chronic diseases.
- Diverse microbiota are synonymous with health, with dietary fiber having a major impact on the composition, diversity, and richness of the microbiome. Thus, accessible carbohydrates are beneficial for the well-being and growth of microorganisms and consequently for the host in this symbiotic relationship.
- Carbohydrates may have mood-enhancing properties, affecting mental and psychological well-being. CHO-rich foods have a negative impact on mood categories, including alertness, tiredness, and higher rates of depression in the long term.
- Carbohydrates, when taken in an adequate amount and in the right balance in the diet, can sustain good mental health.
- Carbohydrate intake can affect the development and prognosis of metabolic disease, as an uncontrolled intake of refined carbohydrates puts individuals at risk of developing metabolic syndrome and subsequently developing metabolic disease.
- The nature of the carbohydrates rather than the amount is the key factor in reducing the risk of cardiovascular disease or in improving the cardiovascular risk markers.
- Complex carbohydrates may modulate insulin-like growth factor binding protein 3 blocking cell proliferation and tumoral growth.
- Fiber intake may modulate gut microbiota and, consequently, their short chain fatty acid production, reducing inflammation and having a positive effect on cancer development.
- Complex carbohydrates and fiber may increase fecal excretion of carcinogens, improving their elimination and reducing their negative effects in organisms.
- Consumption of simple sugars as sweeteners in different sugary beverages is related to asthma due to malabsorption. Formation of pro-inflammatory products between unabsorbed fructose and some dietary proteins, after intestinal absorption, are associated with asthma.
- Nutrition guidelines stablished that carbohydrates make up 40% to 65% of total daily calories to fulfil body energy demands and to reduce the risk of some non-communicable diseases.
- Recommended daily intake of carbohydrates should be based on whole grains.
- Low glycemic index sources and fiber intake should be higher than 25–30 g per day.

## 14. Conclusions

Evidence suggests that carbohydrates, especially fiber, are beneficial for the well-being and growth of gut microorganisms and, consequently, for the host in this symbiotic relationship, producing microbial alterations a negative effect on mental health and different organic systems. In addition, evidence suggests a negative impact of simple carbohydrates and refined carbohydrates on mood categories, including alertness and tiredness, reinforcing a vicious circle. Regarding physical health, sugar intake can affect the development and prognosis of metabolic disease, as an uncontrolled intake of refined carbohydrates puts individuals at risk of developing metabolic syndrome and, subsequently, developing metabolic disease.

## Data Availability

Not applicable.
